# Polymer-Based Electrophoretic Deposition of Nonwovens for Medical Applications: The Effect of Carrier Structure, Solution, and Process Parameters

**DOI:** 10.3390/md19100533

**Published:** 2021-09-23

**Authors:** Ewelina Pabjanczyk-Wlazlo, Nina Tarzynska, Anna Bednarowicz, Adam K. Puszkarz, Grzegorz Szparaga

**Affiliations:** Institute of Material Science of Textiles and Polymer Composites, Faculty of Material Technologies and Textile Design, Lodz University of Technology, Żeromskiego Str. 116, 90-924 Lodz, Poland; nina.tarzynska@dokt.p.lodz.pl (N.T.); anna.bednarowicz@dokt.p.lodz.pl (A.B.); adam.puszkarz@p.lodz.pl (A.K.P.); grzegorz.szparaga@p.lodz.pl (G.S.)

**Keywords:** electrophoretic deposition, biopolymers, hyaluronate, alginate, nonwovens, surface modification, polymeric biomaterials, PLA, micro-CT

## Abstract

Hyaluronate and alginate are non-toxic and biocompatible polymers, which can be used for surface modification and functionalization of many kinds of materials. Electrophoretic deposition (EPD) has several advantages, including its versatility, simplicity, and ability to coat substrates with complex shapes, and is used for the creation of antimicrobial or hydrophobic coatings on metallic biomaterials, among other applications. However, its utilization for applying biopolymer layers on textiles is very limited due to the more complex structure and spatial characteristics of fibrous materials. The aim of this research was to analyze the effects of selected EPD process parameters and the structural characteristics of fibrous carriers on the kinetics of the process and the microscopic characteristics of the deposited layers. The influence of solution characteristics, process parameters, and carrier structures obtained using two different techniques (melt blown and spun-bonded) were analyzed. The morphology and structure of the created deposits were analyzed using scanning electron microscopy and computed tomography, and molecular structure analysis was performed with Fourier Transform Infrared spectroscopy. The surface mass and thickness of fibrous poly (lactic acid)-based carriers were analyzed in accordance with the respective standards. This study serves as a basis for discussion and further development of this method with regard to fibrous materials for medical applications.

## 1. Introduction

Sodium hyaluronate (SH) and sodium alginate (SA) are non-toxic, biocompatible, and biodegradable polymers, which can be used for surface modification and functionalization of many kinds of materials [1]. Sodium hyaluronate, a polysaccharide consisting of N-acetyl-D-glucosamine and D-glucuronic acid units, can be found in all living organisms and is one of the compounds with an identical chemical structure in both bacteria and mammals. Hyaluronate occurs in high concentrations in the skin, joints, and corneas [2]. Its ability to absorb and retain large amounts of water is a characteristic feature, and in tissue engineering, it affects the migration, adhesion, and proliferation of cells [3,4,5]. Moreover, it exhibits characteristic viscoelastic properties [6], which are used in the production of films, fibers, and porous structures, as well as organic/inorganic composites for biomedical applications [7,8]. Hyaluronate coatings have been developed to modify the surface of materials used for prosthetic cartilage production, vascular grafts, and nerve regeneration, among others [9,10,11]. Alginate is a linear copolymer that consists of D-mannuronic and L-guluronic acid blocks connected by β-1,4-glycosidic bonds [12]. Due to its biodegradability, non-toxicity, and biocompatibility, it is also used in tissue engineering and biomedicine [13,14,15] for the fabrication of scaffolds, wound dressings, and fibers.

Electrophoretic deposition (EPD) is a technique used to deposit layers of inorganic, polymeric, metallic, and composite materials. The method is based on the motion of colloidal particles or polymer macromolecules under an electric field, which results in their deposition as layers on the electrode, and the thickness and morphology of the obtained layers can be controlled by adjusting solution characteristics and process parameters [16]. Recently, interest in this technique has greatly increased, especially in industrial applications, due to its high versatility (in regard to the choice of coating materials), the simplicity of the entire process, and the low cost of the equipment and its maintenance. Additional advantages include but are not limited to the short time of deposition, the few constraints on the shape of the substrate (carrier) and thus the ability to coat substrates with complex shapes, and the fact that, in contrast to other advanced deposition techniques, the EPD process can be easily modified for a particular application; it also allows the creation of composite layers [17,18]. For example, deposition can be performed on flat or cylindrical surfaces, or any other surface, and this requires only minor changes in the design of electrodes and their positioning [19]. Among other materials, it is possible to deposit graphene oxide and a graphene oxide/titanium oxide mixture on an aluminum substrate using the electrophoretic deposition process [20,21]. These coatings, additionally subjected to the annealing process, change the hydrophobic properties of the product by reducing the number of oxygen functional groups. As the temperature of the annealing process increases, the contact angle of the modified material increases. The authors also indicate that such materials acquire resistance to corrosion and the harmful effects of chlorine. Additionally, coatings containing graphene oxide and titanium oxide can change from hydrophobic to hydrophilic under the influence of UV radiation.

As a polymer that contains anionic -COOH groups, hyaluronate can be used in the electrophoretic deposition process to obtain a layer of hyaluronic acid (HA) from sodium hyaluronate solutions [22,23]. Similarly, sodium alginate, as an anionic polymer, can also be used in the electrophoretic deposition process, through which layers of alginic acid (AA) can be obtained from sodium alginate solution [24,25]. Electrophoretic deposition has been successfully used to achieve specific biological characteristics of metallic biomaterials (e.g., antimicrobial layers) or to obtain specific surface characteristics (e.g., hydrophobic or antiadhesive coatings) or a few different characteristics. However, knowledge of the EPD of various types of biopolymer layers on fibrous carriers is very limited. The application of this method to textiles is limited due to their more complex structures and spatial characteristics in comparison with metallic biomaterials with different kinds of composite and bulk layers. The available literature on textile electrophoretic deposition focuses on the deposition of conductive polymers, such as polyesters or metal oxides, on fabrics made of conducting polymers. This can find applications in different kinds of sensors, flexible and smart textiles, or flexible supercapacitors [26,27,28,29].

**Figure 1:**
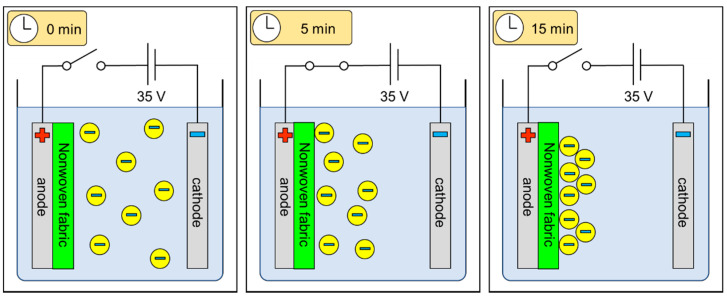
The schematic representation of the electrophoretic deposition process on fibrous structures.

The work presented herein focuses on non-conductive, biocompatible polymer electrophoretic deposition on non-conductive textiles for use in medical and cosmetic applications. The schematic explanation of the electrophoretic deposition process on fibrous structures is presented in Figure 1.

For polymer-based fibrous carriers, in addition to standard factors influencing the process, one should consider the carrier material’s properties, its conductivity, and its structure. All three might in fact hinder the process of deposition due to the barrier imposed by the non-conductive polymeric material of the carrier, which might weaken ion migration. The aim of this research was to analyze the effects of selected electrophoretic deposition process parameters and the structural characteristics of the fibrous carriers on the kinetics of the process and the microscopic characteristics of the deposited layers. In this respect, the following parameters were analyzed: solution characteristics (concentration, conductivity, and surface tension), process parameters (the applied voltage, time, and temperature of deposition), and varying carrier structures obtained using two different techniques (melt blown and spun-bonded). Both processes produce nonwovens with non-uniformly distributed and randomly oriented fibers on the surface and in the fibrous layer, which results in porosity and variable structural parameters, including pores, so their values strongly depend on the site of measurement. It is therefore difficult to predict and control the distribution of individual fibers and their agglomerates, as well as the distribution within the entire layer of the nonwoven [30]. The general difference between the two structures is the obtained characteristics of fibers: melt-blown webs are weaker, bulkier, and thicker than spun-bonded webs [31]. As a result of the uneven structural characteristic of fibrous structures, the expected layer might not be as uniform as that formed by bulk materials (e.g., metallic materials).

The electrophoretic deposition of polymer layers on fibrous carriers is an interesting option for the modification of these materials, as it opens the possibility of a new broad application of this technique. The biopolymer layers can change the characteristics of the surface of such materials, imparting them with favorable properties, such as biocompatibility, desired biological activity, higher or lower surface adherence, and many more. Some constraints stem from the wet method, which might not be useful in certain kinds of applications. In addition, it is important to take into account the relation between the effectiveness of deposition and the structural parameters of the carrier, which can be a very important factor, as shown in the study presented herein. The resulting nonwovens with layers may be very promising for medical, cosmetic, and in general, textile industries. Such deposited nonwovens can serve as wound dressings for patients who have severe burns or are recovering from medical treatments, bedridden patients who require special medical supplies, etc. Such materials could also be successfully used in the cosmetic industry as body care textiles or treatment textiles. Future applications may include utility textiles, e.g., towels with a moisturizing layer or functional textiles used in the production of clothing, which would also have moisturizing functions. This study serves as a basis for discussion and further development of this method with regard to fibrous materials.

## 2. Results

The analysis of the solution characteristics and their influence on the mass of deposits is presented in Table 1. The results confirm that the higher concentration of polymer solutions, which is associated with higher conductivity and surface tension, resulted in higher deposit mass. Differences in the zeta potential of the polymers did not result in differences in the electrophoretic deposition process between sodium alginate and sodium hyaluronate or between sodium hyaluronate of two different molecular weights (variants considered: 80–130 kDa and 1.8–2.0 MDa).

High-molecular-weight sodium hyaluronate (HMW-SH) resulted in a higher zeta potential than low-molecular-weight SH, which may be attributable to the increased supply of ions resulting from the greater supply of carboxyl groups of hyaluronic acid molecules, which can be associated with longer chains. Interestingly, the stability of HMW-SH was better than that of lower-molecular-weight SH, which might be the effect of the increased formation of hydrogen bonds between chains of the polymer or increased electrostatic interactions between ions. Thus, HMW-SH solutions were more stable than the lower-molecular-weight solutions [Cv = 0.48]. In general, sodium alginate solutions had lower stability than sodium hyaluronate solutions. The model research conducted with aluminum foil showed that a solution concentration higher than 1.5% resulted in a gelling effect, which inhibits the migration of ions in the solution and deposition (and has the effect of dip coating with the polymeric solution of the carriers instead of depositing the hyaluronic acid layer). Based on this step, it was possible to select solutions for further studies. In both cases, the solutions with the highest concentration were selected (C = 1.5%).

**Figure 2:**
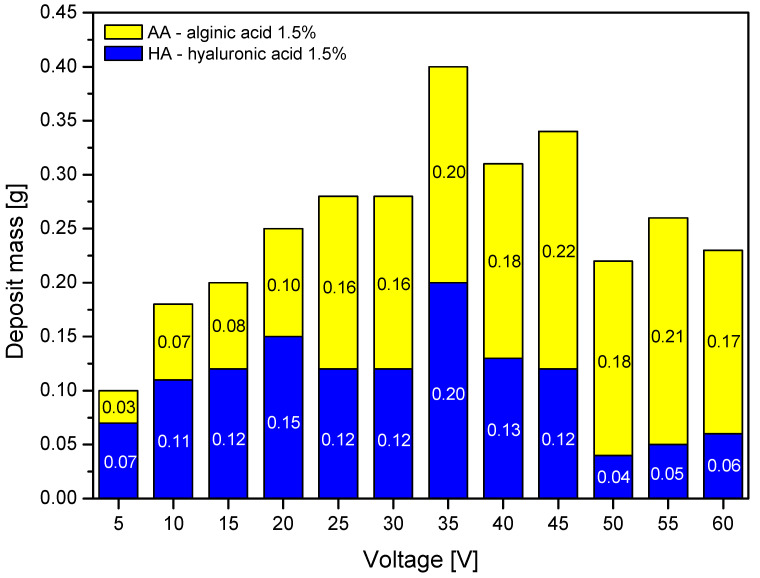
The impact of the voltage on the deposit mass with different variants. Abbreviations: HA—hyaluronic acid; AA—alginic acid.

Further analysis focused on the process parameters. The analysis of the impact of the voltage on the deposit mass is shown in Figure 2.

For both polymers, the deposit mass was the highest at a process voltage of 35 V. When the voltage magnitude exceeded this value, the mass of the hyaluronic acid deposit decreased, while for alginic acid this dependence either was not confirmed, or the decrease was much smaller. This might be caused by excessive charging of the hyaluronic acid molecules and the occurrence of a cross-linking effect in the form of a hydrogen-bonding network among chains, as described in [32], and aggregation, both hindering the mobility of ions towards the carrier.

**Figure 3:**
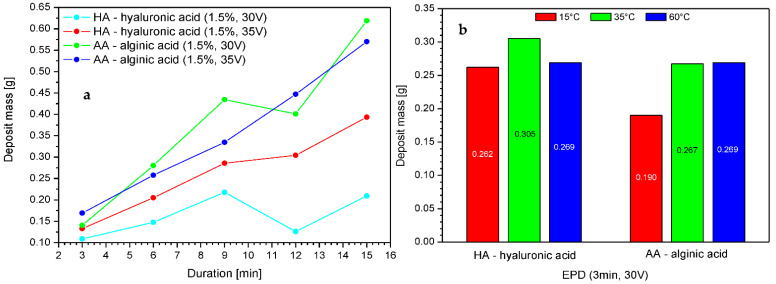
The impact of the voltage, duration of deposition (**a**), and temperature (**b**) on the deposit mass with different variants. Abbreviations: HA—hyaluronic acid; AA—alginic acid.

The influence of the process duration on the efficiency of the electrophoretic deposition process was examined in electrophoretic deposition trials carried out for up to 15 min at 30 and 35 V based on the earlier analysis. The results are shown in Figure 3.

Increasing the time of deposition resulted in a higher deposit mass; however, beyond a threshold of 15 min, the mass dropped. This is connected to the depletion of free ions from the solution constituting the deposit. Alginic acid exhibited slightly higher deposition efficiency as compared to hyaluronic acid, which might be connected to the lower molecular weight of the polymer molecules and better mobility as compared to HMW-HA. The electrophoretic deposition of polymers is largely determined by the physico-chemical equilibrium present in the deposition solution. There are many different interdependent processes taking place, starting from dissociation and migration of ions to the electrodes, followed by deposition in an insoluble form on the electrode, which reduces the number of ions available in the solution, and ending with periodic changes in the pH and conductivity of the solution. This means that the deposition process, especially if it is disturbed by an additional insulator layer, which in our case is a PLA nonwoven fabric, is the result of a subtle balance between all of these processes. Therefore, we suspect that the local decline of deposit mass around 12 min is due to equilibrium processes or may be due to the use of nonwoven fabric obtained from different parts of the sheet. Unfortunately, nonwovens, regardless of the type of production, are characterized by relatively heterogeneous structures, which could, for example, serve to inhibit the process to an extent due to insulation and prevention of deposition. This risk should always be considered when the key criteria are repeatability and the homogeneity of carriers and layers (e.g., for commercial applications).

The effect of temperature can be observed with different intensities in the case of both polymers. A temperature higher than 35 °C either resulted in a decrease in efficiency or did not change the mass of the deposit. Increasing the temperature of the deposition solution affects the electrophoretic transport of ions by influencing different solvent properties, such as viscosity, electric conductivity, and pH, the values of which affect the effectiveness of the process [33]. Therefore, we expect that in the case of hyaluronic acid, high temperature can locally and temporarily change the pH of the solution, which in turn affects the ionic form of the polymer; e.g., by achieving its isoelectric point, when the population of ampholytic molecules (with cationic and anionic functional groups) is at its maximum, and the concentrations of the anionic and cationic species (which migrate to the electrodes and are deposited on the carrier) have the same minimum concentration. In this case, undissociated molecules may also be present. Moreover, the temperature of 60 °C could locally cause the degradation of hyaluronic acid chains, which might hinder the deposition process. Thus, both cases might slightly affect the efficiency of the process when using hyaluronic acid salt solutions.

**Figure 4:**
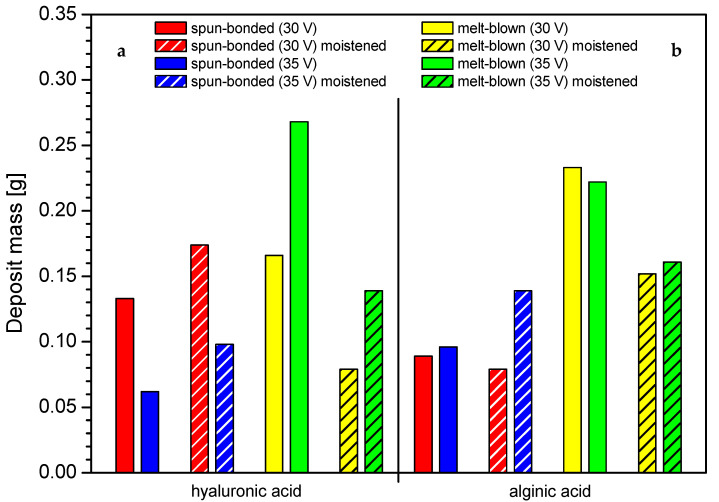
The impact of initial moistening on the deposit mass in different carrier structures: (**a**) hyaluronic acid, (**b**) alginic acid. Abbreviations: SB—spun-bonded; MB—melt blown; HA—hyaluronic acid; AA—alginic acid.

Interestingly, it was observed that the effect of initial moistening could affect the process of deposition, as presented in Figure 4. For the spun-bonded carrier, the moistening increased the deposit mass; this relation was also confirmed for higher voltage. In contrast, for the melt-blown nonwovens, the initial moistening resulted in a decrease in the deposit mass. Wetting affected the nonwovens differently due to their structure. The thickness of melt-blown nonwovens, the structure of which is less dense than that of spun-bonded nonwovens, was significantly reduced, resulting in structure compression under the influence of water. As a result, the deposition solution has difficulty penetrating between the fibers. In contrast, the use of initial moistening facilitated the electrophoretic deposition process in spun-bonded nonwovens, as it might cause small reduction of the surface tension of the nonwoven, thus making its structure more accessible to the penetration of the deposition solution.

**Figure 5:**
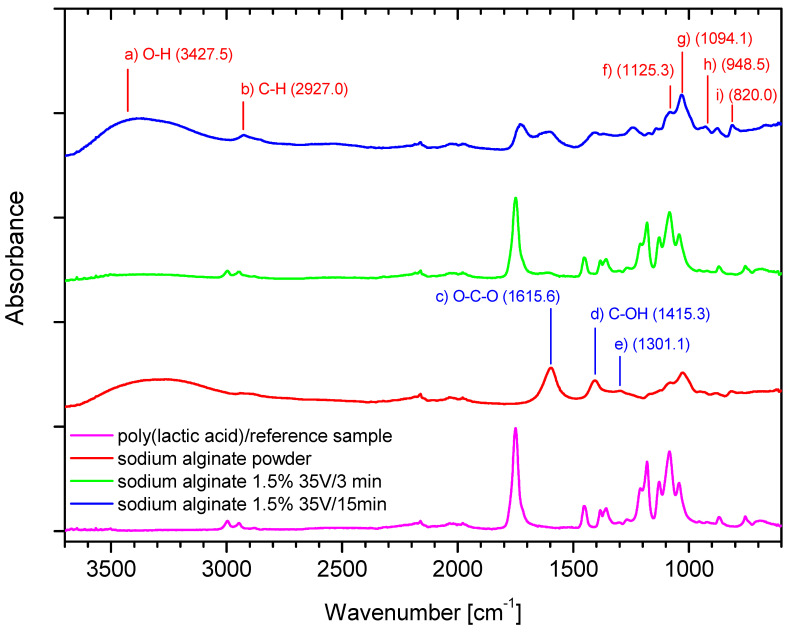
The FTIR analysis: Ref (PLA)—carrier; SA—sodium alginate powder; SA—alginic acid deposits in different conditions.

**Figure 6:**
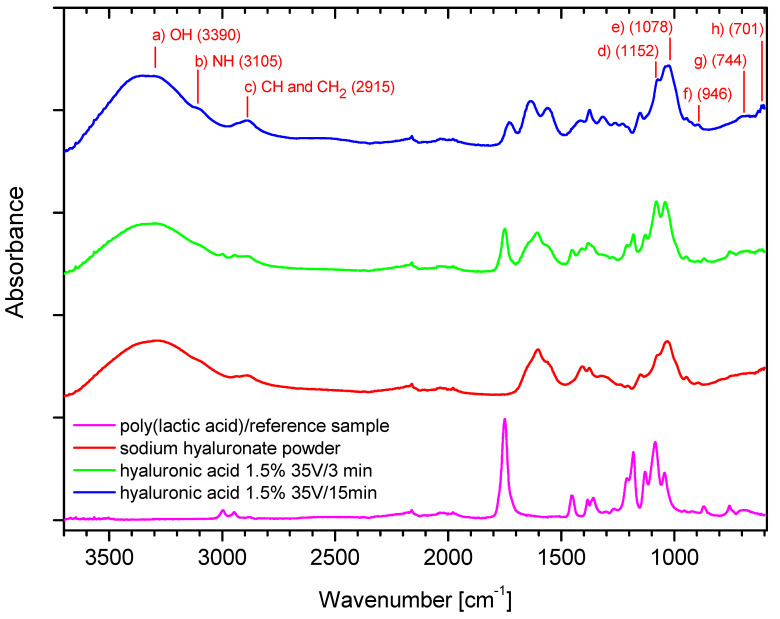
The FTIR analysis: Ref (PLA)—carrier; SH—sodium hyaluronate powder; HA—hyaluronic acid deposits in different conditions.

The FTIR analysis was conducted to confirm the molecular structure of the deposits, as presented in Figure 5 and Figure 6.

The peak at 3427.5 cm^−1^ (a) in Figure 5 can be attributed to hydrogen-bonded O–H stretching vibrations, which is not seen in the poly (lactic acid)/reference sample (without deposit). The weak signal at 2927.0 cm^−1^ (b) in Figure 5 can be assigned to C–H stretching vibrations, and 1615.6 cm^−1^ (c) in Figure 5 can be attributed to the asymmetric stretching of O–C–O groups. The band at 1415.3 cm^−1^ (d) in Figure 5 can be assigned to C–OH deformation vibrations. The right part of the spectrum in Figure 5 presents a few weaker bands at 1301.1 (e) and 1125.3 (f) and the point 1094.1 cm^−1^ (g) in Figure 5 which can be assigned to C–C–H and O–C–H deformations, C–O stretching; and C–O; and C–C stretching vibrations respectively, as discussed in [34,35]. The 950–750 cm^−1^ region applies to uronic and mannuronic acid residues characteristic for alginates. The bands at 948.5 cm^−1^ (h) and at 820.0 cm^−1^ (i) in Figure 5 can be assigned to the C–O stretching vibration of uronic acid and mannuronic acid residues [36].

The peak at 3427.5 cm^−1^ (a) in Figure 5 is less intensive for the sample with shorter time of deposition (3 min, green), as compared to the sample with 15 min of deposition (blue), which might confirm that the longer time of deposition causes the increase in the number of -OH groups referred to the deposited layer. Thus, the intensity and broadness of this peak stem from the larger share of -OH groups in the deposited layer, which results in mass increase obtained in longer time of deposition. The peaks are derived from alginate. The bands c–g in Figure 5 refer to two samples with deposits (green—3 min/35 V and blue—15 min/35 V) in the regions 1700–1800 cm^−1^, 1300–1500 cm^−1^, and 1250–1000 cm^−1^, which cause flattening of the peaks of the poly (lactic acid)/reference sample, which is associated with covering the carrier with a deposit layer. The effect of flattening increases over time of deposition.

The band at 3390 cm^−1^ (a) in Figure 6 can be assigned to -OH groups and is absent in the poly (lactic acid)/reference sample, and the relation between two peaks from samples with deposits depends on the time of deposition. A longer deposition time resulted in a higher intensity of the peak along the whole curve. The band at 3105 cm^−1^ (b) in Figure 6 can be assigned to the -NH stretching band, while that at 2915 cm^−1^ (c) in Figure 6 to -CH symmetrical and -CH_2_ asymmetrical stretching. The 1200–1500 cm^−1^ region can be attributed to deformational vibrations of groups having symmetry, such as H-C-H, CH_2_OH groups, while the 940–1200 cm^−1^ region can be assigned to highly coupled v(CO), v(C-C) and (COOH) vibrational modes, as discussed in [37], and is characterized by a few centered bands at 1152 cm^−1^ (d) in Figure 6, at 1078 cm^−1^ (e) in Figure 6, and at 946 cm^−1^ (f) in Figure 6. The weak band at 744 cm^−1^ (g) in Figure 6 can be assigned to -CH_2_ rocking, and that at 701 cm^−1^ (h) in Figure 6 can be attributed to -CH out-of-plane bending. In case of bands in the 1500–1700 cm^−1^ region, we can observe the intensification of the right arm of the peak for the sample with longer deposition time. The similar relation is apparent in the 1200–1500 cm^−1^ region. Thus, the spectrum course confirms that higher intensity of peaks is associated with longer time of deposition.

The FTIR analysis allowed confirmation of the chemical structure of the deposits for both polymers. The similarity of the spectrum of the samples, namely, sodium hyaluronate powder and samples with deposits (green, blue), might suggest that the deposit is composed also from a sodium salt form of the polymer. It might be caused by the specific helical conformation that hyaluronic acid adopts in aqueous media [38] and the gelation and cumulation of this form at the carrier surface in the preliminary step of the deposition.

**Figure 7:**
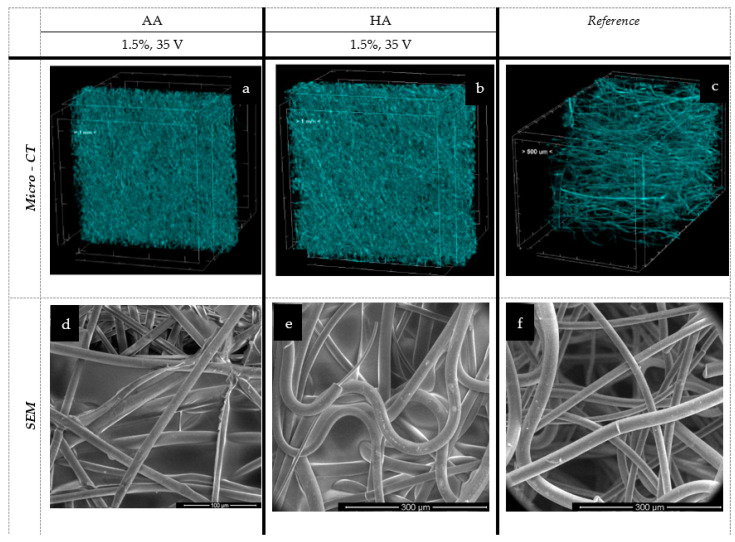
The spatial and microstructure analysis of melt-blown deposits: AA—alginic acid; HA—hyaluronic acid; EPD: t = 3 min, V = 35 V. Scare bars: 100 μm (**d**), 300 μm (**e**), 300 μm (**f**).

**Figure 8:**
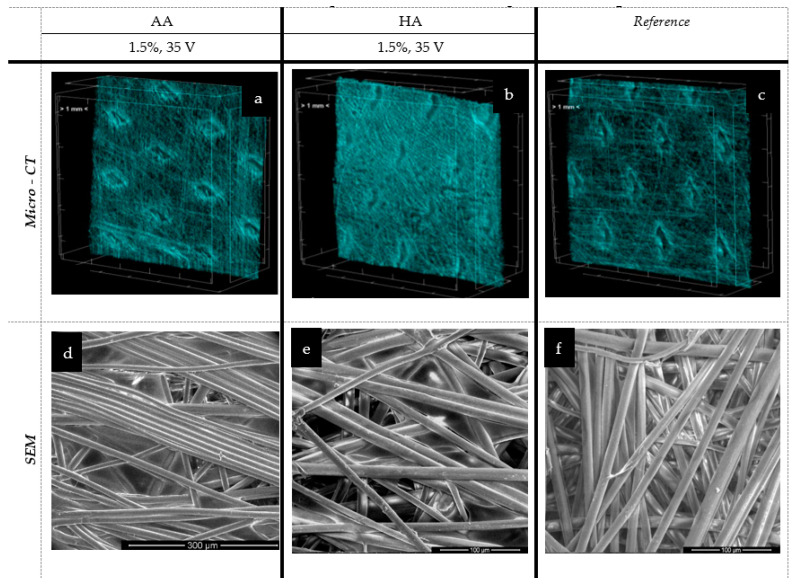
The spatial and microstructure analysis of the spun-bonded deposits: AA—alginic acid; HA—hyaluronic acid; EPD: t = 3 min, V = 30/35 V. Scare bars: 300 μm (**d**), 100 μm (**e**), 100 μm (**f**).

Figure 7 and Figure 8 present the spatial analysis of the deposits carried out with micro-CT and SEM microscopy.

In the case of the melt-blown technique and based on an analysis of Figure 7 and Table 2, the electrophoretic deposition process reduced the thickness of the nonwoven by up to 26.5% as compared to the unmodified nonwoven. Simultaneously, the porosity of the nonwoven decreased by 40% after deposition. The reduction in the thickness of the nonwoven fabric is due to the sticking of the fibers, and thus, there is a compression effect under the influence of water. This relationship is evident for the melt-blown nonwoven due to the large differences in structure compared to the spun-bonded nonwoven, which has a much looser fiber structure and orientation, as confirmed by the comparison of SEM and micro-CT images (Figure 7c and Figure 8c) and their initial greater thickness. For spun-bonded nonwovens, we observe a slight increase (up to 10%) in thickness, which may be associated with the additional polymer layer, with no clear tendency observed in the case of porosity.

In all analyzed melt-blown samples, a deposit layer can be observed with significant phenomena of nonwoven compression and reorientation of fiber filaments. This results in a more chaotic orientation of fibers in comparison to the dominant (horizontal) arrangement of fibers in the entire volume of the reference sample. In the macroscopic view, the deposited samples exhibit dense and inhomogeneous polymer layers that do not cover the outer layer of the carrier but are embedded in its depth. In the case of alginic acid, partial delamination of the layer occurred (the so-called peeling process), which suggests that the created layers may have low durability, an issue that requires further analysis. This means that, as a result of the conducted tests, the layer does not fully cover the outer part of the nonwoven; however, it was possible to obtain it within the outer part of the carrier.

However, a similar situation was observed in the case of spun-bonded sheets. The layer seems to be located in the outer space of the carrier (not literally at the surface), but in this case, the sticking of fibers on the surface part is more visible, and no peeling effect was observed. The spun-bonded carriers are presented in Figure 8.

## 3. Discussion

Electrophoretic deposition strongly depends on the solution characteristics, including the type of polymer and its specific behavior in the solvent, the polymer concentration, and the type of solvent and its accompanying properties, such as pH, surface tension, zeta potential, and conductivity, which together can promote or counteract the deposition process [18]. In this study, only small and comparable concentrations were used. Thus, a 3-fold increase in the concentration (0.5 → 1.5%) of hyaluronic acid resulted in almost a 100% increase in conductivity, a 6-point increase in surface tension, and a 5-fold increase in deposit mass, while for alginic acid, 3-fold, 6-point, and 5-fold increases were obtained, respectively. From the point of view of the effectiveness of the process, a higher polymer concentration resulted in a higher deposit mass. However, it must be taken into account that higher concentrations might result in a gelling effect, high surface tension, and agglomeration of molecules, which can hinder the deposition by preventing ion migration. This effect is mostly seen in hyaluronan solutions, in which concentrations near to 4% create stable gels. Thus, the balance between concentration and effectiveness needs to be considered.

During analysis, the optimal ranges of process parameters were selected, which ensured the highest efficiency of the process. The optimal voltage range was 30–45 V for both polymers, and the optimal deposition time was 3–15 min. The process time should be adjusted to the expected mass of the deposit while considering the concentration of the polymer in the solution. The acid ions are depleted as the process is carried out; thus, it is necessary to create a continuous delivery station for the deposition solutions or to adjust the concentration of the solution for this purpose, taking into account previous conclusions. An increase in voltage resulted in an increase in deposit mass but only up to a particular threshold. Beyond 45 V, the mass dropped, which was more pronounced for hyaluronic acid. The influence of temperature on the deposition process is not clear. Theoretically, an increase in temperature should result in faster ion migration, but it did not have any effect in the case of alginic acid and led to a decrease in the mass of the deposit in the case of hyaluronic acid. It should be added that due to the helical structure of hyaluronic acid in the solution, high temperature can cause local loosening or even degradation of the structure of the chains, which may result in a lower deposit mass than that obtained at, e.g., t = 35 °C. In addition, the increase in the temperature of the deposition can locally and temporarily change the pH of the solution, which in turn affects the ionic form of the polymer, which might slightly affect the efficiency of the process when using hyaluronic acid salt solutions. The presence of characteristic groups in alginic acid and hyaluronic acid was confirmed by FTIR analysis, but in the case of hyaluronic acid, there is no certainty as to whether the acid itself, its sodium form, or both are present in the layer. The analysis also confirmed the effect of the deposition time on the spectrum intensity.

FTIR, microscopic, and microtomographic analyses of the studied nonwovens confirmed that the electrophoretic deposition process results in very irregular deposits, which may be due to the structural heterogeneity of the fiber carriers. Thus, further research is necessary, especially to compare textiles with more uniform structures, such as woven or knitted fabrics. In structural terms, EPD causes significant changes in the structure of the carriers, which raises the question of the expected structural properties of the carrier itself after electrophoretic deposition, e.g., in terms of porosity, thickness, and air permeability. On the other hand, in the case of textiles, the deposition process is affected by the type of carrier; its structure, porosity, conductivity, and thickness; the number, thickness, and orientation of fibers; and surface tension, all of which must be considered.

The produced nonwovens, covered with hyaluronic and alginic acid, could also be used as cell scaffolds. For example, Gorodzha et al. created scaffolds made of nonwoven fabric composed of nanofibers produced by the electrospinning method [39]. The melt-blown and spun-bonded nonwovens described in this article also have a porous structure with a disordered fiber arrangement. In addition, the layer of biodegradable hyaluronic or alginic acid creates another porous structure on the surface that would facilitate the growth of cells on the product.

## 4. Materials and Methods

Poly (lactic acid) (PLA) in the form of granules of density 1.24 g/cm^3^ was purchased from Natureworks LLC (USA). The PLA nonwoven carriers for EPD were prepared by two methods—melt blown and spun-bonded—from PLA Ingeo 6201D and PLA Ingeo 6251D, respectively. The nonwovens were characterized in terms of surface mass, thickness, and fiber diameter, as presented in Table 3. The surface mass of nonwovens was determined in accordance with the PN-EN 29073-1:1994 standard. The samples were acclimatized following the PN-EN ISO 139:2006/A1:2012 standard in a normal climate (air temperature 20 ± 2 °C, relative humidity 65 ± 4%). The thickness of the nonwovens was determined in accordance with PN-EN ISO 9073-2:2002 using a thickness gauge with a DM100 pressure sensor dedicated to flat textiles.

Sodium alginate (SA; Protanal^®^ LF 10/60 L Alginate, Mv = 89 Da) and sodium hyaluronate (SH; Mv = 1.8–2.0 MDa; Mv = 80–130 kDa) were purchased from FMC Corporation and Contipro Biotech, respectively. The dedicated laboratory stand for electrophoretic deposition consists of two electrodes, a container made of synthetic material, a frame for attaching the electrodes, and a generator of voltage. The carriers for deposition were attached to the anode (+). Solutions of SA and SH with concentrations of 0.5–3.0% were prepared with distilled water, stirred for 12 h with a mechanical stirrer, and kept for degassing. The surface tension measurements of deposition solutions were performed with a process tensiometer (Radian Series 300; Thermo Scientific, United Kingdom) using Wilhelmy’s plate method. The conductivity of deposition solutions was measured with a multifunction meter (CX-701; Elmetron, Poland) and conductivity sensor (EC-60) at Tref = 25 °C. The zeta potential of the aqueous solution of polymers was measured with Zetasizer Nano ZS (Malvern Analytical) at a concentration of 0.4 g/100 mL. The applied process voltage was in the range of 5–60 V with 5 V increments. Prior to deposition, a few PLA samples were subjected to initial moistening with 0.5 mL of distilled water per sample using a simple atomizer to enhance the effectiveness of the deposition. The EPD process was conducted for 3–15 min and at T = 15, 25, 35, or 60 °C. The samples were dried at room temperature. The effectiveness of the process was measured as the mass gain of the carrier with deposition over time in the selected conditions. Preliminary studies were performed on a research model using aluminum foil in order to eliminate disruptions to the process due to the attachment of the nonwoven to the electrode (and predicted weakening of the voltage and ion migration force), and the most optimal deposition solution characteristics (polymer concentration, surface tension, and conductivity) were selected.

The surface morphologies of deposits and reference samples were analyzed using a scanning electron microscope (FEI NOVA NanoSEM 230) equipped with a field-emission electron gun (FEG) and with micro-CT (SkyScan 1272, Bruker, Kontich, Belgium) using the following scanning conditions: X-ray source voltage 50 kV, X-ray source current 200 µA, and rotation 180° with a rotation step of 0.2°. The pixel size was 5.5 µm for spun-bonded nonwovens and 6.5 µm for melt-blown samples. The molecular structure analysis of bulk sodium salts and unmodified and modified nonwovens was performed with an FTIR spectrophotometer (Thermo Scientific, Nicolet 6700), with a resolution of 4 nm and 64 scans methodology described previously in [40].

## 5. Conclusions

The study presented herein confirms that electrophoretic deposition can be used for the modification of fibrous materials. However, a few important findings from the study need to be considered. The electrophoretic deposition is a result of a subtle balance between all processes taking place in the deposition solution. In addition to the traditional process, EPD on fibrous structures requires the analysis of the impact of the carrier structure on the course and efficiency of the process.

Based on a preliminary analysis, it was possible to select the optimal characteristics of the deposition solutions for further studies. Higher concentrations of polymer solutions, associated with higher conductivity and surface tension, resulted in higher deposit mass. For both polymers, the highest solution concentration was selected (C = 1.5%), and due to a possible gelling effect, it should not be increased much beyond this value. The next step included the optimization of process parameters and selection of the most optimal conditions of EPD. The analysis of the voltage influence revealed that the most optimal process voltage was 35 V for both polymers, which was associated with the highest deposit mass. Increasing the time of deposition also resulted in higher deposit mass but only up to 15 min, as the mass dropped after this threshold. Temperatures higher than 35 °C either resulted in a decrease in efficiency or did not change the mass of the deposit. The effect of initial moistening could affect the process of deposition by inducing the sticking of fibers and the compression of the nonwoven, which could in turn result in difficulties in the penetration of the deposition solution between the fibers. This effect was more noticeable for melt-blown nonwovens, in which the moistening adversely affected the deposit mass, while for spun-bonded, it increased the deposits masses.

The micro-CT analysis revealed that in the case of the melt-blown technique, EPD reduced the thickness of the nonwoven by up to 26.5% as compared to the unmodified nonwoven. Simultaneously, the porosity of the nonwoven decreased by 40% after deposition. Conversely, for spun-bonded nonwovens, we observed a slight increase (up to 10%) in thickness, which may be associated with the additional polymer layer. The micro-CT and SEM analysis confirmed the presence of the deposited layer in all analyzed samples, showing the same phenomena of nonwoven compression and reorientation of fiber filaments as melt-blown nonwovens. The presence of characteristic groups for alginic acid and hyaluronic acid was confirmed by FTIR analysis. The analysis also revealed that the electrophoretic deposition process resulted in very irregular deposits, which may be due to the structural heterogeneity of the fiber carriers. Thus, further research is necessary, especially to compare textiles with more uniform structures, such as woven or knitted fabrics.

Based on this study, the most optimal conditions were selected for both polymers and both types of nonwovens, allowing the highest mass of the deposit to be obtained; the selected conditions were concentration (C) = 1.5%, voltage = 35 V, time = 15 min, T = 35 °C, and optional moistening (only for spun-bonded nonwovens).

## Figures and Tables

**Table 1:** The comparison of surface tension and conductivity of deposition solutions with different polymer concentrations in reference to the deposit mass. Abbreviations: SH—sodium hyaluronate; HA—hyaluronic acid; SA—sodium alginate; AA—alginic acid.

Polymer	Concentration [%]	Conductivity [mS/cm]	Surface Tension [mN/m]	Deposit Mass [mg] EPD: 3 min, 35 V	Zeta Potential ^1^ [mV]; Cv [%]
SH/HA 1.8–2.0 MDa	0.5	0.97	73.09	45	-
	1.0	1.07	76.33	117	-
	1.5	2.06	80.42	195	−92.1; 0.07
SH/HA 80–130 kDa	-	-	-	-	−24.6; 0.48
SA/AA 89 Da	0.5	1.60	69.15	39	-
	1.0	2.60	72.51	125	-
	1.5	4.34	75.38	198	−51; 0.36

^1^ The zeta potential measurements were taken for a concentration of 0.4 g/100 mL.

**Table 2:** The microtomographic characteristics of the selected structural parameters of the nonwovens with deposits. HA—hyaluronic acid; AA—alginic acid.

Sample	Thickness [mm]	Porosity [%]
Melt blown (reference)	4.90	82.9
1.5% HA (35 V)	1.70	62.5
1.5% AA (35 V)	1.30	49.1
Spun-bonded (reference)	0.26	58.2
1.5% HA (35 V)	0.28	34.8
1.5% AA (35 V)	0.29	65.6

**Table 3:** Poly (lactic acid) nonwoven structural characteristics according to the method of fabrication.

Method	Surface Mass [g/m^2^]	Thickness [mm]	Fiber Diameter [μm]
Melt blown (MB)	174.48	0.24	61.61
Spun-bonded (SB)	62.20	0.27	9.50

## Abbreviations

AA; SA	alginic acid; sodium salt of alginic acid, sodium alginate
CT; micro-CT	computed tomography; micro-computed tomography
EPD	electrophoretic deposition
FTIR	Fourier transform infrared spectroscopy
HA; HMW-SH; SH	hyaluronic acid; high-molecular-weight sodium hyaluronate; sodium hyaluronate
MB	melt blown
PLA	poly(lactic acid)
SEM	scanning electron microscopy
SB	spun-bonded
